# The Effects of Mindfulness-Based Strategies on Perceived Stress and Psychobiosocial States in Athletes and Recreationally Active People

**DOI:** 10.3390/ijerph19127152

**Published:** 2022-06-10

**Authors:** Selenia di Fronso, Claudio Robazza, Réka Zsanett Bondár, Maurizio Bertollo

**Affiliations:** 1 Behavioral Imaging and Neural Dynamics (BIND) Center, 66100 Chieti, Italy; c.robazza@unich.it (C.R.); zsanett.bondar@unich.it (R.Z.B.); m.bertollo@unich.it (M.B.); 2 Department of Medicine and Aging Sciences, “G. d’Annunzio” University of Chieti-Pescara, 66100 Chieti, Italy; 3 Department of Neuroscience, Imaging and Clinical Sciences, “G. d’Annunzio” University of Chieti-Pescara, 66100 Chieti, Italy

**Keywords:** body scan, dynamic and static strategies, emotions, MBSR, mindful yoga, sitting meditation, sport/physical activity, stress perception

## Abstract

The mindfulness-based stress reduction (MBSR) programme is gaining increasing attention in sport and physical activity domains. This programme comprises three meditation practices: mindful yoga, body scan, and sitting meditation. In this study, we aimed to examine the effects of a dynamic (mindful yoga) strategy and a static (a combination of body scan/sitting meditation) strategy on participants’ psychobiosocial states (PBS), perceived stress (PS) and mindfulness levels in athletes and recreationally active (RA) people. Thirty-four participants (athletes = 18; RA participants = 16) were assigned to a dynamic intervention strategy, and another 34 (athletes = 19; RA participants = 15) were assigned to the static intervention strategy. Before the intervention, after the intervention and three weeks later, the Italian versions of the PBS scale, the PS scale and the Mindful Attention Awareness scale were administered. RM-(M)ANOVAs revealed that intervention strategies improved functional PBS, reduced PS and enhanced mindfulness levels in both athletes and RA participants after the intervention (*p* < 0.001, η_p_^2^ = 0.605). However, improved functional PBS after the intervention (*p* < 0.001; *d* = 0.62) and stable PS levels at follow-up (*p* = 1) were observed mainly in athletes. The findings reinforce the view of the importance of the body as a means to improve emotional and health processes, and support the use of mindfulness strategies in sport to enhance individuals’ well-being.

## 1. Introduction

Mindfulness-based practices generally derive from ancient Buddhist meditations, such as Vipassana and Zen meditations, and include psychological interventions such as dialectical behavioural therapy, acceptance and commitment therapy, brief mindfulness induction (e.g., a single or few short-term sessions that teach a mindful approach to the present moment), and mindfulness-based stress reduction (MBSR) [1,2]. Overall, it has been demonstrated that mindfulness-based practices can lead to increased levels of self-reported mindfulness, enhanced psychological functioning and well-being (e.g., [3]), improved emotion regulation (e.g., [4]) and reduced anxiety and depression in clinical populations (e.g., [5,6]). Moreover, the literature indicates that mindfulness interventions can ameliorate self-regulation [7], reduce rumination, and facilitate emotional control [8] in healthy adults.

Over the last years, mindfulness-based practices have gained increasing attention, even in the sport domain (e.g., [9,10]). This is likely due to their impact on athletes’ mental aspects (e.g., focused attention, mental toughness) leading to performance enhancement [11]—see for example, performance enhancements in sports, such as shooting and dart throwing [12,13]—and their beneficial effects on athletes’ well-being. MBSR is currently one of the most commonly adopted practices in this domain (e.g., [14,15]). MBSR [16,17] generally consists of three main meditation practices [18], which include (1) mindful yoga, in which participants cultivate mindful awareness of the body while it is moving, stretching, or holding a position; (2) body scan, in which participants sequentially and non-judgmentally focus their attention on parts of the body and body sensations; and (3) sitting meditation, in which participants focus their attention on their breathing, body sensations, sounds in the environment, and their stream of thoughts and emotions [19].

Overall, this kind of standardised training has been proven to reduce anxiety, depression, pain and stress (e.g., [20]). With regards to athletes, MBSR has been found to improve psychological well-being among retired football players [15] and to enhance athletic coping skills and sleep quality in female collegiate rowers [14]. Moreover, injured athletes can benefit from including MBSR in the sport rehabilitation process to increase their pain tolerance and awareness [21]. Additionally, in the physical activity context there is a growing interest in the effects of mindfulness in general, and MBSR in particular. For instance, a school-based intervention including mindfulness training was shown to produce medium positive effects on mental and social health, and small effects on physical activity levels in adolescent girls [22]. Meier and colleagues [23] also found that a combination of exercise and MBSR was successful in increasing general well-being and physical activity participation in healthy adults. However, to the best of our knowledge, research comparing the effects of mindfulness between athletes and people involved in a physical activity context, such as recreationally active (RA) participants, is rare, especially with regards to MBSR interventions.

Furthermore, notwithstanding the increasing number of studies on the potential benefits of MBSR among athletes and people practising physical activity, in the sport and physical activity domains there is still scant research aimed at disentangling the effects of the meditation practices included in the programme. In this regard, intervention dismantling studies could be useful to better understand which components of a treatment programme induce specific changes [24]. For example, in a dismantling study by Sauer-Zavala et al. [19], undergraduate participants reported significant improvements in self-compassion and psychological well-being regardless of the intervention strategies. The authors noticed that (1) mindful yoga increased psychological well-being to a greater extent than the other two types of practice (i.e., sitting meditation and body scan); (2) both sitting meditation and mindful yoga were superior to body scan in improving emotion regulation; and (3) sitting meditation was associated with a greater increase in a non-evaluative stance toward observed stimuli than body scan. To date, however, no study has distinguished the effects of a dynamic mindfulness-based strategy focused on body movements from those of a static strategy.

Drawing on the findings of the aforementioned study, we conducted a randomised dismantling investigation to examine (1) the effects of a dynamic mindfulness-based strategy (i.e., mindful yoga with a specific focus on mindful movements, conducted with compassionate acceptance toward each thought, feeling, memory, emotion and bodily sensation) [25] on participants’ psychobiosocial states (PBS), perceived stress (PS) and mindfulness levels in comparison to a static mindfulness-based strategy (i.e., a combination of sitting meditation and body scan); and (2) the differential effects of these strategies in athletes and recreationally active participants on the above mentioned variables. Overall, the examination of PBS and PS is based on the mounting evidence on the positive effects of MBSR and mindfulness in general on emotions and stress (e.g., [14]), and on recent study findings showing the negative effects of the pandemic on athletes’ psychological conditions (e.g., [26,27]). Given the compelling evidence in support of mindful-movement interventions for reducing stress and related outcomes [28,29], we hypothesised a more impactful effect of the dynamic strategy on both athletes and recreationally active participants. Moreover, considering that athletes generally cope better with stress [26,30] than recreationally active people, we expected that the latter group of participants would benefit more from both intervention strategies.

## 2. Materials and Methods

### 2.1. Participants

The eligibility criteria for participating in our study included participants at least 18 years old with no previous experience of mindfulness-based strategies. In addition, participants had to regularly complete at least 150 to 300 min of moderate-intensity activity per week or 75 to 100 min of vigorous-intensity activity per week, plus muscle-strengthening activities 2 or more days per week, thus meeting the World Health Organization (WHO) minimum activity guidelines [31]. Alternatively, they had to practise a sport discipline and be engaged in competitions. Seventy-two people signed an on-line informed consent before the testing procedure. Four out of 72 did not meet the inclusion criteria and were excluded from our research project. Thus, 68 participants (women = 42) aged from 22 to 54 years (M = 32 ± 10) voluntarily took part and completed the study. Thirty-seven out of 68 participants were involved in a variety of different individual or team sport disciplines such as gymnastics (*n* = 9), track and field (*n* = 2), swimming (*n* = 3), golf (*n* = 7), volleyball (*n* = 10), handball (*n* = 3), football (*n* = 3) and competed at different levels (i.e., international, *n* = 2; national, *n* = 24; regional, *n* = 11). Thirty-one out of 68 participants did not practice sport but met the WHO minimum activity guidelines as described above. Accordingly, participants were classified as athletes (*n* = 37) and recreationally active (RA; *n* = 31) people.

### 2.2. Measures

The participants completed an online survey consisting of a battery of questionnaires, including (a) a demographic information form; (b) the Psychobiosocial States Scale (PBS-S) [32]; (c) the Perceived Stress Scale (PSS) [33], Italian version (I-PSS) [34], and (d) the Mindful Attention Awareness Scale (MAAS) [35], Italian version [36].

#### 2.2.1. Demographic Information Form

After completing the informed consent, participants were asked to provide information about their gender, age, and sport/physical activity involvement at the time of the survey. Specific examples of questions included in this form are “Do you practice sport? Which one? At what level do you compete? If you do not practice sport, are you used to completing at least 150 to 300 min of moderate intensity activity per week, or 75 to 100 min of vigorous-intensity activity per week, plus muscle-strengthening activities 2 or more days per week?”

#### 2.2.2. Psychobiosocial States Scale

Athletes’ psychobiosocial states were measured through the Psychobiosocial States scale (PBS-S) developed by Robazza and colleagues [33] in Italian. The 15 items used in this scale measure trait-like experiences and derive from the 20-item profiling approach [37] for an individualised assessment of athletes’ experiences associated with successful and poor performances. This assessment is based on the assumption stemming from the Individual Zones of Optimal Functioning model [38,39] that athletes commonly experience several performance-related pleasant and unpleasant feelings, some of which can foster sport performance while others can disrupt it. The PBS-S scale assesses 8 functional modalities (i.e., pleasant affective, anger, cognitive, motivational, volitional, bodily-somatic, motor-behavioural, operational) and 7 dysfunctional modalities (anxiety, cognitive, motivational, volitional, bodily-somatic, motor-behavioural, and operational) scored in intensity on a scale ranging from 0 (nothing at all) to 4 (very much). Each item consists of 3–4 synonym descriptors, except the pleasant-affective modality, which contains 5 descriptors. For both groups of participants, we modified the directions as follows: “How did you feel during the last month in relation to your training activities?” Cronbach’s alphas in the study by Robazza and colleagues [33] were 0.78 for functional PBS and 0.74 for dysfunctional PBS scales.

#### 2.2.3. Italian Perceived Stress Scale

Participants’ perceived stress was evaluated through the Italian 10-item version of the Perceived Stress Scale (IPSS-10) [34]. This scale evaluates one’s general stress levels related to events that occurred in the month before the detection, and also assesses current levels of experienced stress. It consists of 6 negatively stated items (e.g., “In the last month, how often have you been/felt unable to control the important things in your life?”) and 4 positively stated items (e.g., “In the last month, how often have you been/felt on top of things?”) scored on a 5-point Likert scale ranging from 0 (never) to 4 (very often). Total scores are calculated after reversing positive items scores and then summing up all scores, with a total score ranging from 0 to 40. A higher score indicates a higher level of perceived stress. Cronbach’s alpha for the Italian version was 0.74 [34].

#### 2.2.4. Mindful Attention Awareness Scale

The 15 items of the Mindful Attention Awareness Scale (MAAS) [35], Italian version [36], were used to capture participants’ mindfulness levels and experiences. The items of this scale refer to experiences of acting automatically (e.g., “I do job or task automatically, without being aware of what I’m doing”) and without paying attention to the present moment (e.g., “I find it difficult to stay focused on what’s happening in the present”). We modified the stem of items as follows: “In the last month: I did job or task automatically…”. Participants could rate the 15 items using a 6-point Likert-type scale ranging from 1 (almost always) to 6 (almost never). Higher scores indicate higher levels of mindfulness. Cronbach’s alpha for the Italian version was >0.80 [40].

### 2.3. Procedure

Participants were recruited by phone, email, and adopting a snowball sampling technique [41]. In particular, using our informal networks, we started with a known group of people and some of them recruited other participants among their acquaintances. As mentioned above, mindful yoga, sitting meditation and body scan were categorised as a dynamic intervention strategy (i.e., mindful yoga) and a static intervention strategy (i.e., sitting meditation/body scan). Participants were randomly assigned to the two study conditions, which were held in separate time slots but at similar times of the day. They were provided with general information on the purposes of the study while specific information was omitted. The enrolment in one condition precluded participation in the other condition. In detail, 34 participants (athletes *n* = 18; RA participants *n* = 16) were randomly assigned to the dynamic intervention strategy and 34 (athletes *n* = 19; RA participants *n* = 15) to the static intervention strategy.

Participants in both the dynamic and static condition attended a 10-session programme (lasting approximately one hour for 2 sessions a week) delivered across an intervention period of one month and a half (from the beginning of October to the middle of November 2021). Each mindful yoga session (dynamic strategy) started with mindful walking exercises followed by yoga poses and yoga pose transitions, and ended with a discussion about participants’ experiences and possible difficulties encountered during the sessions. Sitting meditation/body scan sessions (static strategy) included body parts and breathing awareness and focusing attention exercises (e.g., paying attention to the sounds in the environment) and concluded with the same discussion as in the dynamic intervention strategy. Participants in both conditions were provided with links to websites containing the exercises they experienced in the sessions and were required to continue the practice on their own once a day between sessions [19]. All sessions were led by a MBSR expert and conducted online due to COVID-19 pandemic restrictions. Participants were also encouraged to practise in a quiet and safe environment to maintain their activities and comfort. More details on the programme will be made available from the authors upon reasonable request.

The questionnaires were administered through an online survey platform (i.e., Google Forms) five days prior to the beginning (T0) of the programme, five days after the conclusion (T1), and three weeks later (T2). The follow-up assessment (i.e., T2) was conducted to examine the degree to which effects seen shortly after the implementation of the interventions, persisted over time. The day of the first assessment (i.e., T0), participants signed the informed consent and completed the demographic information form before accessing the questionnaires. The study was approved by the Ethics Committee for Biomedical Research of Chieti-Pescara University (ID richiam7px) and was undertaken in compliance with the Declaration of Helsinki and the international principles governing research on humans.

### 2.4. Analysis

An initial screening of the data did not reveal missing values or multivariate outliers [42]. The examination of histograms, skewness, and kurtosis of the variable scores did not show substantial deviation from normal distributions. The reliability of the measures was ascertained using Cronbach’s alpha. However, as alpha has been criticised as an inappropriate measure of internal consistency reliability [43], we also used McDonald’s omega [44]. Both indices were calculated using the SPSS software (Version25.0; IBM, Armonk, NY, USA).

To assess changes in participants’ functional and dysfunctional PBS, PS and mindfulness scores from pre- to post-interventions and at the follow-up, and examine the effect of the two intervention strategies, two mixed between-within repeated measure analysis of variance were performed. Specifically, we performed a repeated measures multivariate analysis of variance (RM-MANOVA) 3 (time: pre- vs. post-interventions vs. follow-up) × 2 (group: athletes vs. RA participants) × 2 (strategy: dynamic vs. static) on functional/dysfunctional PBS and PS. Regarding mindfulness scores, we performed a RM-ANOVA 3 (time) × 2 (group) × 2 (strategy). In the follow-up ANOVA, Bonferroni correction was used for pairwise comparisons. The sphericity assumption was evaluated using the Mauchly test. Greenhouse–Geisser correction for degrees of freedom was applied in case of non-sphericity. In the ANOVAs, effect sizes were calculated using partial eta square (η_p_^2^) [45], with 0.01, 0.06, and 0.14 considered small, medium, and large effects, respectively. In the case of multiple comparisons, effect sizes were calculated using the Cohen’s *d* [46], for which 0.20, 0.50, and 0.80 are considered small, medium, and large effects, respectively. The significance level was set at 0.05, and statistical analyses were performed using STATISTICA software (Version12; StatSoft, Inc., Tulsa, OK, USA).

## 3. Results

Cronbach’s alpha, McDonald’s omega values, and descriptive statistics are provided in Table 1 and Table 2. The PBS-S, I-PSS and MAAS scales showed acceptable or good reliability and internal consistency values (see Table 1).

**Figure 1:**
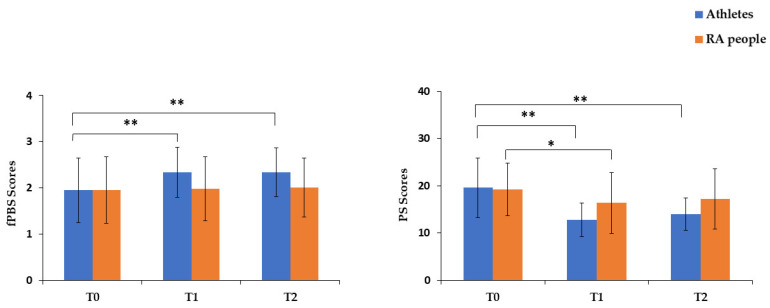
Time × Group interaction in functional psychobiosocial states (fPBS) and perceived stress (PS) in athletes and recreationally active (RA) people. Note: Error bars represent standard deviations. * *p* < 0.05; ** *p* < 0.001. Athletes’ fPBS: T0 vs. T1 Cohen’s *d* = 0.62; T0 vs. T2 Cohen’s *d* = 0.63. Athletes’ PS: T0 vs. T1 Cohen’s *d* > 1; T0 vs. T2 Cohen’s *d* > 1. RA people’ PS: T0 vs. T1 Cohen’s *d* = 0.46.

RM-MANOVA yielded a significant main effect by time, Wilk’s λ = 0.395, *F*(8, 57) = 10.894, *p* < 0.001, η_p_^2^ = 0.605, Power = 1, and by time × group interaction, Wilk’s λ = 0.713, *F*(8, 57) = 2.869, *p* = 0.009, η_p_^2^ = 0.287, Power = 0.917. A significant main effect by group was also found, Wilk’s λ = 0.822, *F*(4, 61) = 3.291, *p* = 0.017, η_p_^2^ = 0.178, Power = 0.809. No significant main effect by strategy (*p* = 0.975), strategy × group (*p* = 0.230), time × strategy (*p* = 0.227) and time × strategy × group (*p* = 0.740) was found. Follow-up univariate ANOVA showed significant differences by time in functional PBS, *F*(1.714, 109.671) = 10.975, *p* < 0.001, η_p_^2^ = 0.146, Power = 0.981. A significant effect by time × group in functional PBS, *F*(1.714, 109.671) = 7.027, *p* = 0.002, η_p_^2^ = 0.099, Power = 0.890, was also observed, as well as a significant effect by group in dysfunctional PBS, *F*(1, 64) = 9.414, *p* = 0.003, η_p_^2^ = 0.128, Power = 0.856. In particular, functional PBS significantly increased (*p =* 0.001, *d* = 0.32) after the intervention programme and their levels also remained high at follow-up (*p =* 0.002, *d* = 0.35), with no significant differences between T1 and T2 (*p =* 1). However, functional PBS scores only increased in athletes, with significant differences between T0 and T1 (*p* < 0.001, *d =* 0.62) and between T0 and T2 (*p* < 0.001, *d* = 0.63). No significant difference was observed between T1 and T2 (*p* = 1). Moreover, compared to athletes, RA participants showed significantly higher (*p* = 0.003, *d* = 0.33) dysfunctional PBS (see Table 2 and Figure 1).

Considering PS, follow-up univariate ANOVA yielded significant differences by time, *F*(1.974, 126.334) = 40, 383, *p* < 0.001, η_p_^2^ = 0.387, Power = 1, and by time × group interaction, *F*(1.974, 126.334) = 7.818, *p* = 0.001, η_p_^2^ = 0.109, Power = 0.946. No significant differences emerged by strategy (*p* = 0.790), strategy × group (*p* = 0.420), time × strategy (*p* = 0.296) and time × strategy × group (*p* = 0.580). In detail, PS significantly decreased after the intervention programme (*p* < 0.001, *d* = 0.86) and follow-up (*p* < 0.001, *d* = 0.69), with no significant differences between T1 and T2 (*p* = 0.186). In RA participants, perceived stress significantly decreased after the intervention programme but slightly increased at follow-up. Thus, we observed significant differences between T0 and T1 (*p* = 0.014, *d* = 0.46), but not between T0 and T2 (*p* = 0.258) or between T1 and T2 (*p* = 1). Similarly, with regards to the athletes, perceived stress significantly decreased after the intervention programme and slightly increased at follow-up (see also Figure 1). However, we observed significant differences between T0 and T2 (*p* < 0.001, *d* > 1). We also found significant differences between T0 and T1 (*p* < 0.001, *d* > 1), but not between T1 and T2 (*p* = 1) (see Table 2).

Considering mindfulness levels, RM-ANOVA yielded significant differences only by time, *F*(1.594, 101.992) = 26.035, *p* < 0.001, η_p_^2^ = 0.289, Power = 1. No significant differences emerged by time × group (*p* = 0.069), strategy (*p* = 0.814), strategy × group (*p* = 0.066), time × strategy (*p* = 0.756) and time × strategy × group (*p* = 0.713). In particular, mindfulness levels significantly increased after the intervention programme but decreased at the follow-up. Specifically, we found significant differences between T0 and T1 (*p* < 0.001, *d* = 0.77), between T0 and T2 (*p* < 0.001, *d =* 0.53), and between T1 and T2 (*p* = 0.020, *d* = 0.32) (see Table 2).

## 4. Discussion

The aim of the current study was to examine the effects of a dynamic mindfulness-based strategy in comparison to a static mindfulness-based strategy on participants’ PBS, PS and mindfulness levels. The differential effects of these strategies in athletes and RA participants on the same variables were also considered.

Firstly, findings revealed that mindfulness-based strategies, regardless of their typology, had a beneficial effect on participants’ psychological conditions. Additionally, the increased mindfulness scores after the intervention suggest that the two strategies were effective and properly delivered. Overall, our findings are in line with previous research on the effects of mindfulness training on stress, emotions and awareness levels in the sport domain. For example, Vidic and colleagues [47] found that a mindfulness intervention decreased perceived stress levels in a U.S. Women’s NCAA Division I Basketball Team, while Jones and colleagues [14] observed that an 8-week MBSR intervention was associated with lower perceived stress scores, higher awareness levels and improved psychological well-being in collegiate rowers. Our findings are also partially consistent with the reduction in competitive anxiety and physiological markers of stress levels found in elite Wushu athletes following an 8-week mindfulness-based intervention [48].

Although it is suggested that the practice of mindful movements, as in mindful yoga (dynamic strategy), leads to substantial improvements in awareness (i.e., awareness of sensations, of the present moment, attention, mind wandering, intention and non-judgment) [49], in our study we did not observe differences in the strategies adopted. The lack of differences between the effects of the dynamic and the static mindfulness-based strategies likely reflects the utmost importance of the attention to body sensations and accompanying feelings that may help people adapt better to the environment and improve their capacity to cope with stress (e.g., [27]). This kind of attention is indeed induced not only by the dynamic strategy through the practice of mindful yoga, but also by the static strategy, especially through the practice of body scan. Our findings also strengthen the idea that while practising mindfulness through dynamic or static strategies, the body (and a mindful contact with it) represents a valuable, positive resource and the place where people mainly experience deep states of calmness and positive emotions [50]. Furthermore, the improvements in PBS, PS, and mindfulness levels reported in our dismantling study are consistent with those found in studies evaluating MBSR in its entirety [51,52]. On the other hand, our findings are somewhat at odds with those of Sauer-Zavala and colleagues [19], who found between-strategies differences in individuals psychological functioning over time. This discrepancy could be due to the different categorisation of the strategies we adopted or to the fact that Sauer-Zavala et al. [19] implemented a shorter intervention protocol. Early differential changes (e.g., emotion regulation) observed in individuals’ psychological health elicited by mindful yoga may not persist in a longer protocol. For instance, in our study, participants might have gained acceptance over body feelings from repeated body scan strategies and sitting meditation, which may have contributed to more functional emotions.

When considering differences by group, we noticed that RA participants were mostly characterised by higher dysfunctional PBS levels, probably because they experience a higher intensity of cognitive and somatic anxiety compared to athletes [26,53]. As for the specific effects of mindfulness-based intervention strategies over time, we observed that our strategies generally had a positive effect on functional PBS and PS, and this effect, despite a decrease in mindfulness levels, was also maintained at follow-up. This finding suggests that both groups likely continued to practise mindfulness after the intervention given its beneficial effects. However, a guided and supervised practice is crucial to maintain high levels of awareness [49]. On the other hand, this also reflects that the effects seen shortly after the implementation of the interventions could only partly persist over time and reinforces the notion that sport and physical activity in general, are essential to maintain positive emotions and reduce stress perception [54]. Furthermore, our outcomes underlined that the interventions, through a non-judgmental and acceptance-based stance towards every kind of feelings and emotions, likely facilitated emotional stability and reinforced functional PBS (e.g., [14]).

Secondly, when considering the interaction between time and groups of participants, we noticed that post interventions, functional PBS only increased in athletes. Additionally, we observed that PS levels remained fairly stable in athletes, while RA participants showed increased level of PS at follow-up. This is likely because RA participants are generally worse at dealing with stress and less effective in regulating emotions than athletes [26,30]. Furthermore, RA participants were experiencing more dysfunctional PBS, which could have hampered their emotional stability and/or their capacity to self-regulate through mindfulness activities. Moreover, athletes in general are required to constantly control and manage their emotions under different conditions of training and competition, and therefore they learn how to deal with stressors [55]. Additionally, sport environments and organisations usually provide services to help athletes not only improve their performance and achieve their goals, but also to overcome their psychological challenges. This could have amplified the differences between athletes and RA people.

Some limitations of the current study should be acknowledged and addressed in future research. For example, given the pandemic and the general difficulty of delivering interventions, we could only rely on a purposive sample and a relatively small sample size. Thus, future studies should envisage protocols that include larger samples in order to obtain more generalisable findings. Additionally, we did not consider participants’ personality traits and facets of mindfulness. Accordingly, future intervention protocols should also account for the interrelationship among personality traits, facets of mindfulness and psychobiosocial states [56]. Furthermore, mid-term measurements may shed light on early differential changes in psychological parameters elicited by various mindfulness-based strategies. Moreover, we did not consider the precise amount of moderate- or vigorous-intensity activity that each RA participant practised in a week. This parameter, as well as the athletes’ specific competitive level, the type of sport practised and the inclusion of a control group that do not practise any mindfulness-based strategies should be taken into account in further investigations. These variables may indeed help to obtain more reliable results and a more nuanced overview on the effects of mindfulness practice in the sport and physical activity domains. In addition, future studies should assess individuals’ skin conductance level, heart rate, heart rate variability, and electroencephalographic activity to provide a clearer idea of the psychophysiological mechanisms that underlie the effects of mindfulness-based activities in athletes and recreationally active people.

## 5. Conclusions

Although our hypotheses were only partly confirmed, the current study still affirms the importance of mindfulness-based intervention strategies in the sport and physical activity domains. In particular, the findings shed light on the functional combination of sport and mindfulness-induced effects on stress and emotions, and reinforce the crucial role of body-related mindfulness activities included in a MBSR programme (e.g., mindful yoga and body scan). Moreover, the findings highlight that a guided and supervised practice can facilitate individuals’ awareness. Although dynamic strategies in general can be easier to implement, especially with athletes, in this investigation, static strategies seem to be equally effective in reducing PS and improving functional PBS. This paves the way for further research on the topic (for example, see di Fronso and Bertollo’s paper about Yoga Nidra) [57].

From a practical point of view, not only during pandemic or other stressful or unique situations, athletes and RA people can benefit from both dynamic and static mindfulness-based intervention strategies to strengthen their psychological well-being. By practising mindful yoga, mindful walking, yoga nidra, body scan, etc., individuals could be better prepared for potential changing environments and reduce their perceived stress levels. Moreover, they could increase their mindful awareness and develop distinct interoceptive awareness skills, including identifying, accessing, and appraising internal bodily signals, which are seen as crucial components for emotion regulation [27]. Overall, the body-related activities mentioned above may represent those behavioural techniques able to reintroduce feelings of positive affect in individuals’ lives [19]. Importantly, mindfulness practice in the sport domain may also improve motor skills or promote psychological pathways (e.g., decrease in anxiety levels or dysfunctional PBS in general) that reduce the negative impact of emotions on performance outcomes [12]. Of note, since the effects of the mindfulness strategies seem to be less impactful and enduring in RA participants, longer (and supervised) programmes should be recommended.

## Figures and Tables

**Table 1:** Cronbach’s alpha (α) and McDonald’s omega (ω) values of psychobiosocial states, Italian perceived stress, and mindful attention awareness scales before intervention, after intervention and follow-up.

Scale	Time	α	ω
dPBS	T0	0.86	0.87
	T1	0.83	0.83
	T2	0.85	0.85
fPBS	T0	0.88	0.88
	T1	0.85	0.85
	T2	0.85	0.85
PS	T0	0.76	0.76
	T1	0.74	0.70
	T2	0.80	0.78
Mind	T0	0.82	0.83
	T1	0.83	0.84
	T2	0.82	0.83

Note. dPBS = dysfunctional Psychobiosocial States; fPBS = functional Psychobiosocial States; PS = Perceived Stress; Mind = Mindfulness; T0 = Five days prior to the intervention; T1 = Five days after the end of the intervention; T2 = Follow-up three weeks after the end of the intervention; *N* = 68.

**Table 2:** Means and standard deviations of psychobiosocial states, Italian perceived stress, and mindful attention awareness scales before intervention, after intervention and follow-up by type of strategy and group.

Scale	Time	Athletes	RA People	Dynamic	Static
dPBS	T0	0.86 (0.67)	1.12 (0.75)	1.03 (0.66)	0.93 (0.77)
	T1	0.59 (0.39)	1.05 (0.72)	0.74 (0.51)	0.86 (0.69)
	T2	0.58 (0.40)	1.02 (0.70)	0.81 (0.58)	0.75 (0.62)
fPBS	T0	1.95 (0.70)	1.95 (0.72)	1.98 (0.74)	1.93 (0.68)
	T1	2.34 (0.54)	1.98 (0.69)	2.16 (0.57)	2.19 (0.70)
	T2	2.34 (0.53)	2.01 (0.64)	2.22 (0.49)	2.16 (0.70)
PS	T0	19.56 (6.33)	19.16 (5.58)	19.54 (5.73)	18.82 (6.21)
	T1	12.75 (3.55)	16.35 (6.44)	14.73 (5.19)	14.05 (5.55)
	T2	13.94 (3.45)	17.16 (6.37)	15.20 (5.51)	15.61 (4.96)
Mind	T0	4.26 (0.73)	3.96 (0.76)	4.10 (0.78)	4.15 (0.73)
	T1	4.95 (0.37)	4.48 (1.11)	4.72 (0.58)	4.75 (1.02)
	T2	4.82 (0.35)	4.12 (0.71)	4.54 (0.54)	4.47 (0.70)

Note. RA = Recreationally active participants; dPBS = dysfunctional Psychobiosocial States; fPBS = functional Psychobiosocial States; PS = Perceived Stress; Mind = Mindfulness; T0 = Five days prior to the intervention; T1 = Five days after the end of the intervention; T2 = Follow-up three weeks after the end of the intervention; *n* Athletes = 37; *n* RA participants = 31; *n* Active Strategies = 34; *n* Passive Strategies = 34; *N* = 68.

## Data Availability

All the data are provided within the text; the data will also be available upon request to all interested researchers by contacting the corresponding author.
